# Pain management among Dominican patients with advanced osteoarthritis: a qualitative study

**DOI:** 10.1186/s12891-016-1075-y

**Published:** 2016-05-17

**Authors:** Amy Yu, Christopher A. Devine, Rachel G. Kasdin, Mónica Orizondo, Wendy Perdomo, Aileen M. Davis, Laura M. Bogart, Jeffrey N. Katz

**Affiliations:** Orthopedic and Arthritis Center for Outcomes Research, Brigham and Women’s Hospital, 75 Francis St., Boston, MA 02115 USA; Harvard Medical School, 25 Shattuck Street, Boston, MA 02115 USA; Universidad Iberoamericana, Av. Francia 129, Gazcue, Santo Domingo Dominican Republic; Toronto Western Research Institute, University Health Network, 399 Bathurst Street, Toronto, ON M5T 2S8 Canada; Division of General Pediatrics, Department of Medicine, Boston Children’s Hospital, 300 Longwood Avenue, Boston, 02115 USA; Department of Orthopedic Surgery, Brigham and Women’s Hospital, 75 Francis St., Boston, MA 02115 USA; Department of Epidemiology, Harvard School of Public Health, 677 Huntington Ave., Boston, MA 02115 USA; Division of Rheumatology, Immunology, and Allergy, Brigham and Women’s Hospital, 75 Francis Street, Boston, MA 02115 USA

**Keywords:** Osteoarthritis, Total joint replacement, Pain management, Qualitative, International

## Abstract

**Background:**

Advanced osteoarthritis and total joint replacement (TJR) recovery are painful experiences and often prompt opioid use in developed countries. Physicians participating in the philanthropic medical mission Operation Walk Boston (OpWalk) to the Dominican Republic have observed that Dominican patients require substantially less opioid medication following TJR than US patients. We conducted a qualitative study to investigate approaches to pain management and expectations for postoperative recovery in patients with advanced arthritis undergoing TJR in the Dominican Republic.

**Methods:**

We interviewed 20 patients before TJR about their pain coping mechanisms and expectations for postoperative pain management and recovery. Interviews were conducted in Spanish, translated, and analyzed in English using content analysis.

**Results:**

Patients reported modest use of pain medications and limited knowledge of opioids, and many relied on non-pharmacologic therapies and family support to cope with pain. They held strong religious beliefs that offered them strength to cope with chronic arthritis pain and prepare for acute pain following surgery. Patients exhibited a great deal of trust in powerful others, expecting God and doctors to cure their pain through surgery.

**Conclusion:**

We note the importance of understanding a patient’s individual pain coping mechanisms and identifying strategies to support these coping behaviors in pain management. Such an approach has the potential to reduce the burden of chronic arthritis pain while limiting reliance on opioids, particularly for patients who do not traditionally utilize powerful analgesics.

## Background

Arthritis is a leading cause of long-term pain worldwide [1]. Osteoarthritis, in particular, is the most common joint disorder in the US, affecting more than 27 million people [2]. Pain is the cardinal symptom and a major determinant of functional disability and reduced quality of life in patients with osteoarthritis [3, 4]. As there are no effective disease-modifying therapies for the treatment of osteoarthritis, many patients experience inexorable progression of joint damage and attendant pain, ultimately opting for total joint replacement (TJR) surgery [5].

In developed countries, pain management for arthritis is often multimodal, involving a combination of both pharmacologic and non-pharmacologic therapies [6–8]. Patients undergoing TJR experience pain in the period leading up to surgery as a consequence of their underlying joint disease and during postsurgical recovery as a result of the extensive surgical disruption of osseous and soft tissues. Opioids are often used to alleviate preoperative pain in patients with advanced arthritis and are virtually always used for postoperative pain management [7]. In addition to rising concerns about prescription opioid addiction, misuse, and diversion [9–11], chronic opioid dependence in the preoperative setting may put patients at greater risk for complications, and postoperative challenges with pain control and rehabilitation [12]. Yet unrelieved acute pain may become chronic pain, leading to prolonged recovery and increased risk for physical, psychological, and social consequences [13, 14]. As a result, effective pain relief remains a challenging issue in developed countries.

The global prevalence of arthritis has prompted proliferation of philanthropic programs that aim to alleviate the burden of arthritis and associated pain. Operation Walk Boston (OpWalk) is one such program, which has offered TJR to economically disadvantaged patients in the Dominican Republic since 2008. Prior studies have indicated that OpWalk patients experienced substantial preoperative pain as measured by the Western Ontario McMaster Universities Osteoarthritis Index [15]. Nevertheless, OpWalk physicians have observed that Dominican patients undergoing TJR require fewer opioid analgesics during the postoperative period as compared to US patients undergoing the same surgeries. Specifically, these patients appear to request fewer opioids and report an improved recovery experience when their medications are minimized. While prior research has investigated arthritis pain management in developed countries [16, 17], few studies have examined pain management and expectations for TJR recovery for arthritis patients in the developing world, where opioids may not be readily available or affordable [13, 14]. Thus, we conducted a qualitative study to explore the attitudes and beliefs of pain management and expectations for postoperative recovery in patients with advanced arthritis undergoing TJR in the Dominican Republic.

## Methods

### Setting

The Dominican Republic occupies the eastern two-thirds of the Caribbean island of Hispaniola. Of its 10.3 million citizens, approximately 41.1 % live below the poverty line. The per capita gross domestic product is $12,800, of which health care expenditures account for 5.4 % [18]. Although publically funded health care provides basic primary and emergency care for all citizens, the majority of the population cannot afford additional health insurance, and thus lacks access to advanced elective treatments such as TJR [19]. To help address this unmet medical need, the OpWalk Boston team, consisting of approximately 50 health care workers, travels annually to Hospital General de la Plaza de la Salud in Santo Domingo to perform total knee and hip replacements on 40–50 qualifying patients.

### Participants

Participants for this study were chosen from the 40 patients in OpWalk Boston 2015. These individuals learned of the program through advertisement, word of mouth, or physician referral. All patients were low-income and unable to pay for the procedure. The criteria for selection into OpWalk included symptomatic, radiographically advanced knee and/or hip arthritis, stable medical status, and willingness to stay in the Santo Domingo area for 2 weeks after surgery for postoperative visits. Patients for this study were sampled pre-operatively because their perceptions of arthritis pain management and surgery may be altered in the post-operative state. All patients recruited understood that participation in the study was optional and would not impact their upcoming surgery. Due to timing constraints, not all patients enrolled in OpWalk were approached for interviews. For each of the four consecutive days that the study was conducted, participants were selected randomly, to minimize selection bias, from those who had not yet completed surgery. Interviewers met at the end of each day to discuss common preliminary themes observed during the interviews and to determine if data saturation was met. We limited the sample to 20 patients because we reached a point of saturation at which completed interviews revealed commonalities and no longer produced new themes and insights. Consent for participation and publication was obtained from all patients. All study activities were approved by the institutional review board at Brigham and Women’s Hospital.

### Procedures

Three trained interview teams, each comprised of one US student and one medical student from Universidad Iberoamericana, performed 20 preoperative interviews over 4 days during OpWalk Boston 2015. Interviews were conducted in Spanish and followed a moderator’s guide to ensure consistency of content (Table 1). This guide was developed and reviewed by a multinational, multidisciplinary team of investigators to refine questions for clarity and cultural sensitivity. Topics were open-ended and included pain self-care and care seeking behaviors, and expectations for postsurgical pain and recovery. The semi-structured interview allowed patients the freedom and flexibility to elaborate on their answers or raise new discussion topics [20]. Interview teams met each evening to consider revisions to the moderator’s guide. Interviews were conducted in private rooms, audiotaped, transcribed verbatim into Spanish and English by a translation company. All patient identifiers were removed from interview transcripts.

**Table 1:** Moderator’s Guide for Interviews

Topic	Question
Arthritis pain	- Tell me about your arthritis pain. Can you remember the first time you knee/hip started to hurt? Can you describe what your pain feels like? - How have you managed pain related to your arthritis? - How has your arthritis pain impacted your life? - To what extent do you feel you are able to control your arthritis pain?
Pain self-care	- What do you do when you experience pain? Why? - Who do you tell about your pain? Why? - What do you do when someone comes to you with pain? - What types of treatments do you use for your pain? Tell me about your beliefs that influence your use of treatment. - What works best for you to treat your pain? - What do you do when you get a headache? Toothache? Sprain or broken bone?
Pain care-seeking	- Have you ever seen a doctor for pain? If yes, how do doctors ask you about your pain? What do they do for your pain? - What do you know about pain medications? - Have you tried any medications for pain? How did they work? When do you take pain medication? - Do you know the term “opioids?” If so, what do you know about opioids? Have you ever used them? - Do you know the term “morphine?” If so, what do you know about opioids? Have you ever used them?
Postoperative expectations and pain management	- What do you expect the surgery to be like? Do you expect it to be painful? - What do you think postsurgical recovery will be like? Do you expect that it will be painful? - How do you expect your pain to be treated after surgery?

### Data analysis

English transcripts were analyzed using content analysis, a method that classifies textual data into themes to make valid inferences from the text [21]. Two investigators (AY and CAD) initially performed an open reading of five randomly selected interview transcripts to assess basic thematic content. The reviewers met to discuss themes emerging from the data and developed a preliminary coding scheme. A second open reading session was performed using five additional randomly selected interview transcripts to confirm the themes. A final coding scheme was generated consisting of ten codes and 32 subcodes representing five themes. Codes were defined as categories of words, which were subsequently organized into shared themes. One investigator (AY) reviewed five more transcripts to ensure that no further themes emerged. One investigator (AY) then re-coded all 20 interview transcripts using the finalized coding scheme. Quotations were extracted and organized by corresponding themes. Throughout the analysis, Dominican clinicians (MO and WP) were consulted to ensure the themes represented were consistent with patient interviews and cultural context. Furthermore, the Dominican clinicians verified that culturally specific aspects of Dominican language were not misinterpreted when translated into English.

To ensure the reliability of the analysis, another investigator (CAD) coded five randomly selected transcripts using the final coding scheme. The inter-rater agreement among the two reviewers (AY and CAD) in the assignment of specific themes to particular quotations was assessed using Cohen’s Kappa coefficient. The Kappa was 0.86 (95 % CI = 0.80–0.92), reflecting excellent agreement [22]. The investigators discussed and resolved discrepancies.

## Results

Of the 40 patients participating in OpWalk 2015, 20 were approached for preoperative interviews and all consented. Of the interviewees, 16 (80 %) were women and the median age was 62.5 years (range 20–79). Seven received total hip replacements (1 bilateral), and 13 received total knee replacements (5 bilateral). Ten participants were unemployed, seven were self-defined housekeepers, two were retired, and one was employed as a teacher. The sex and age distributions of those who participated were similar to those who did not participate (84 % female, median age 65 years).

### Overview of findings

Patients discussed a range of pharmacologic and non-pharmacologic approaches to pain management, from traditional Western medications to prayer and relaxation. For many, strong religious convictions greatly influenced their ability to cope with painful experiences. Patients spoke powerfully of strength and endurance as means of overcoming pain. Although most patients expected surgical pain, they were prepared to cope with it and were confident that the union of doctors and God would yield a successful surgery. In the following sections, representative quotations for each theme are presented throughout the text and in Table 2.

**Table 2:** Representative quotations from interviews (*n* = 20)

*Pharmacologic management*
“I control [the pain] a lot of times. Sometimes I can’t control it, and then I go to the analgesics” (female, age 75). “If the pain is really bad, I take medication. But I try not to take medication every day” (female, age 55).
“Sometimes the rheumatologist treated me with Methotrexate. But after about 20 days, my liver got inflamed, and I automatically stopped taking it. And I’ve never gone back to using it” (female, age 32). “They [friends] tell me that [diclofenac] does a lot of damage. And I almost never take it” (female, age 79). [Referring to why she limits medication use] “There are times you take certain medications, and you feel it in your stomach” (female, age 59).
“I stopped taking Prednisone because [friends] say it causes liver damage” (female, age 73).
*Non-pharmacologic management*
“I sit for a while, I relax, and that’s it” (female, age 63). “I try to forget about the pain, and a lot of the time I do get better, when I forget about the pain” (male, age 20). “I do therapy with the electric blanket, a wet towel from the freezer, and cold water” (female, age 75).
“When the pain comes, I only tell God because he is the one I acknowledge” (female, age 52). “Often when I’m in pain, there are times I don’t want to take painkillers. So I put myself in God’s hands, and I pray a lot” (female, age 59). [Referring to how she copes with painful episodes] “I go to God, because everybody has to go to God” (female, age 75). “I do believe that God has helped me, because I didn’t have the strength. I asked God, and He gave it to me. So there is the belief that God exists. It’s a belief that God will give me the strength, and He gave it to me” (male, age 20).
*Endurance*
“I learned how to endure it” (female, age 74). “Once the problem happens to you, you have to keep going and get up. Because when you are in pain, as long as you can, you endure it” (female, age 52). “If you have something, you have to fight it. You can’t throw it behind you. You have to accept things” (female, age 75). “Sometimes if you have a positive outlook, things get better” (female, age 59). “When you are in pain, you endure it. But when the pain is really bad, you take a painkiller” (female, age 72).
*Family support*
“I have to depend on my husband to be able to do something” (female, age 55). [Referring to relationship with family] “Lots of bonding, lots of support, lots of strength from them, especially from my parents” (female, age 44). “I’ve learned several things from my family with their support and assistance. Being here, I have gotten more support than if I had been doing well, you could say. They [family] always think of you first, they always want [you] to get better” (male, age 20). “He [brother-in-law] is the one that helped me get the operation” (female, age 79). “They [my family] always went to the doctor. I follow my mother’s example” (female, age 59).
*Postoperative expectations and pain management*
“It will be painful, because it’s an object put inside me. I have to overcome [the pain]” (female, age 75). “At least the time to come [after surgery] is going to relieve the pain” (female, age 76). “All recoveries are painful. They are never easy. It all depends on your mentality” (female, age 75). “I trust in God and the doctor that it will be a success” (male, age 74). “I trust in both God and the surgeons, since I know they are very good and all. You being the team that you are, everything will work out well. That’s really what we hope for, and God willing, that this pain will go away; it’s really tiresome” (female, age 32). “The people at home are lifting me up in prayer, so that I will get well [following surgery]. With God’s help and the doctors” (female, age 75).

### Pharmacologic management

Most patients reported experience with pain medications, yet personal preferences varied from regular self-administration to sparse use only when absolutely necessary. Those who reported taking medications tended to rely on non-steroidal anti-inflammatory drugs (NSAIDs) including diclofenac and ibuprofen. A few patients described using steroid injections or topical menthol creams to alleviate pain. When asked about specific analgesia terminology, only two of 20 preoperative interviewees were familiar with the term “opioid.” One recognized it as a derivative of opium: “it’s a plant, a legume” (male, age 62). The other described using tramadol for a short period of time, during which she experienced side effects including “intense euphoria” and sleeplessness. Half of the interviewees recognized the term “morphine,” of which one recalled using the drug following a prior orthopedic surgery. Most who recognized the drug name understood its role as an analgesic, but none knew of others who had used it previously. One patient remembered reading about morphine: “It’s in novels, books from that time period… in the Old West” (male, age 62). Patients were further asked if they would take morphine if available. Many expressed limited knowledge of the drug, but stated they would follow a doctor’s order if morphine was prescribed. On the other hand, one patient stated: “I don’t want to go to such an extreme that I have to use [morphine]” (female, age 75).

In discussing the frequency and timing of preferred medication use, few patients described routine administration of painkillers. For the majority of patients, fear of side effects was a major concern that caused them to use medications sparingly. One patient shared: “I can’t take them, or use them, because I get side effects. Painkillers help my problem but I run the risk [of side effects]” (female, age 72). Another patient discussed the desire to prolong the interval between each dose of medication: “Sometimes I take a painkiller. Sometimes. Not every time, but maybe every 15 days or so. Because when I take a medication, it makes my stomach hurt” (female, age 63). Patients often credited their knowledge of side effects to conversations with family and friends: “People told me that [diclofenac] was a little harmful for the kidneys… So I decided to cut it in half to limit its effect on my body” (male, age 20). Some also cited fear of addiction as a reason to limit medications: “I didn’t want to take much medication because when you start taking them every time something hurts, you get addicted… I didn’t want to do that to myself” (female, age 63).

### Non-pharmacologic management

Almost all patients described using therapies other than painkillers to reduce pain. Some discussed relaxation and distraction as a means to forget about their pain: “When it hurts a lot, I lie down for a while” (female, age 74). Another patient shared: “When I am in a lot of pain, I always try not to focus on the pain, I look for some way to distract my mind” (female, age 32). Four patients found exercise or physical therapy to be valuable complements to medication. For them, moving their joints relieved pain rather than instigating it: “I have to stand up and move around, because when I’m moving, the pain goes away” (female, age 75). Seven individuals rubbed ointments and creams on their joints, and three discussed using cold compresses or ice to alleviate acute pain.

Among the most common approaches to pain management were religion and prayer. Many who held strong religious beliefs understood pain as a part of life and an experience to endure: “Pain is a reaction that you have to live with, because if God sent it, you have to endure” (female, age 61). Fifteen patients discussed the role of faith in overcoming pain. Patients formed partnerships with God through prayer, and God rewarded them with strength to bear the pain: “I say: ‘Lord, if you think I deserve more, send it to me. Just give me the strength to continue to endure it.’ And I stand up like nothing happened. When I pray to the Lord, the peace I feel inside is so strong, it makes me forget about the physical pain” (female, age 32). One patient depicted her conversations with God: “I put myself in God’s hands and I pray. I stay strong, because God says: ‘You must be strong.’ If God is with me, who can be against me?” (female, age 61). Another shared: “Often when I’m in pain, I pray to God. God is number one, because He is the one who directs our lives. He knows when someone may be in pain, when it can be taken it away” (female, age 59). For some individuals, God not only provided strength to endure pain, but also granted power to use medicine: “God takes away the pain. God takes your hand, and you put menthol or something on it, and then it calms down” (female, age 73).

### Endurance

Although religion afforded many the strength to endure, patients also found strength within themselves. One individual described her experience with pain as a battle: “I keep fighting, I fight, fight, fight. Because the pain won’t defeat me, I am going to defeat the pain” (female, age 75). Another patient cited the power of positivity to provide strength: “I always try to smile. Because for me, it’s one of the things that helps me endure it, to overcome the pain” (female, age 32). Patients described the desire to live as normally as possible, in spite of their pain. A retired physical therapist stated: “I never stopped doing things, never, never, never skipped a case in the middle of the day” (female, age 75). When speaking of pain management, many believed endurance to be a primary means of coping, and used medication as a last resort.

### Family support

Patients also emphasized the importance of social support in coping with pain. Family members are often primary points of contact when they experience pain. Patients cited various examples of emotional and physical support from family, such as consoling, aiding in doctor visits, performing day-to-day tasks, and providing therapies. One described: “You go to family when you have something, and the family tries to take you to the doctor, give you painkillers” (female, age 63). Moreover, family traditions often dictated how pain was managed. Referring to pain medications she uses, one patient stated: “We use things [medications] from the past. You always carry those things your grandparents told you” (female, age 59). Finally, patients often mentioned family members as the ones who helped them enroll in OpWalk.

### Postoperative expectations and pain management

When asked about postoperative pain and recovery, the majority of patients interviewed understood that surgery itself could cause pain, but they were not afraid of the possibility. Patients reported being so accustomed to arthritis pain that surgical pain would not bring new challenges: “Since I’ve had so much pain, that kind of [surgical] pain doesn’t scare me. I can get over that easily. They open it, sew it, and that will get better, because skin heals. I think my leg pain was worse” (male, age 20). Patients were asked about long-term pain relief; many expressed hope that surgery would diminish their arthritis pain, if not completely relieve it: “I know it’s to help with the pain, to get rid of the pain” (female, age 73). In anticipating postsurgical pain management, all patients expected to take less pain medication each day, and most expressed the desire to end pharmacologic therapy as soon as possible.

For many patients, their commitment to God provided reassurance and confidence that surgery would be successful: “I believe in God, because with God first, I believe I will be okay. I trust in Him that everything will turn out well” (female, age 59). Some discussed how divine intervention played a role in their acceptance into OpWalk, suggesting that surgery was God’s gift: “I have been asking God to give me an opportunity, and He gave it to me… We are poor, we don’t have any resources. Just God. What God provides” (female, age 61). When asked about coping with pain during surgical recovery, many patients conveyed confidence that God would cure their pain. One stated: “This pain I have now, I won’t have it tomorrow, because God is going to heal me” (female, age 63).

Patients also expressed a great deal of trust in doctors, with many discussing both God and doctors as key players in their journey through surgery: “I hope that with the Lord’s strength and the doctors, I will get well” (female, age 75). Another shared: “I trust in you and God. You will help me to be able to walk” (female, age 63). For one patient, God and physicians are closely intertwined, as God empowers surgeons to perform operations for the betterment of humankind: “You all are doctors, His instruments, because He [God] is the one that does it. He uses you as instruments to do things” (female, age 59).

## Discussion

This is the first qualitative study, to our knowledge, that investigates pain management among patients with advanced osteoarthritis in a developing nation. We interviewed 20 patients before TJR to understand their approaches to pain management and expectations for postoperative recovery. Patients reported modest use of medications, possessed limited knowledge of opioids, and employed a range of non-pharmacologic coping strategies including prayer, distraction, and relaxation. Many patients turned to religion as an explanatory model of and coping mechanism for pain. They understood pain as God’s will and an experience to be endured, equipping them to expect surgical pain and persevere through postoperative recovery. Our interviews revealed that many patients with chronic pain exhibited a great deal of trust in powerful others, expecting God and doctors to eventually cure their pain through surgery.

Prior research has illuminated ways in which individuals respond to setbacks and stressors. Lazarus and Cohen described stressors as internal or external environmental demands which upset balance, requiring action to restore equilibrium [23]. In the transactional model, an individual first appraises a stressor as a harm (negative) or a challenge (positive) and then evaluates if he or she possesses the coping resources to deal with that stressor [24]. This model has been studied in a variety of disease stressors, including pain in patients with musculoskeletal disorders [25, 26]. Negative appraisals, such as catastrophizing, have been correlated with more intense pain experiences, heightened disability, and emotional distress, while positive appraisals have been associated with physical and psychological adjustment [27, 28]. In our study, many patients acknowledged pain as a stressor to be endured, defeated, or cured; they tended not to catastrophize their pain experience. Our interviews suggest that in the face of pain and surgery, patients exhibited an external locus of control, placing the fate of their pain in the hands of powerful others including God and surgeons [29]. In response to both chronic (arthritis) and acute pain (surgery), patients adopted a wide repertoire of coping resources, which included strong ties to God, family support, and minimal reliance on medications. Moreover, patients emphasized strength and endurance in combating pain, coping behaviors which were often rooted in religious beliefs. Similarly, in a study of cancer pain management in Hispanic patients in the US, pain was often approached with stoicism, and endurance was seen as a source of personal strength and pride [30].

Pain coping strategies have been researched extensively in rheumatologic conditions in developed countries. These strategies are often classified as active coping such as physical activity, or passive coping involving rest and withdrawal from activities [31]. Studies in the US have shown that patients with osteoarthritis use both active and passive strategies, with passive strategies predicting higher levels of pain and disability [25]. In our sample, Dominican patients consistently employed active coping practices in attempts to manage their illness and associated pain, through strategies such as religion, physical therapy, and distraction. We also note a contrast in pain management approaches observed in our cohort as compared with individuals in the developed world. Although non-pharmacologic interventions are often recommended to patients in developed countries, a study of 205 US veterans with osteoarthritis revealed relatively low use of non-pharmacologic therapies, including exercise, physical therapy and dietary/herbal supplements [32]. In contrast, nearly all patients in our study emphasized the importance of identifying non-pharmacologic resources and strategies to control pain. Understanding ways in which our findings could be applied to improving the utilization of such non-pharmacologic approaches in the developed world is an important area for future investigation.

We also suggest core socio-cultural differences between the US and Dominican Republic may help to explain our observations. In the US, the emphasis on pharmacological accessibility, direct-to-consumer advertising, and patient activation yields a health care model that empowers patients to request powerful pain medications, including opioids [33]. In contrast, our interviews suggest that in the Dominican Republic, shared cultural beliefs on religion, pain as God’s will, and endurance generate a different attitude towards pain management, one in which reliance on medication is often reduced. Certainly, this observation is further informed by disparities in medication access, as limited access to analgesics such as opioids remains a challenging issue in many developing nations [13, 14].

Furthermore, our findings elucidate the significant role religion can play in each aspect of the pain experience. Our subjects’ comments suggest that religion and pain are closely intertwined, as individuals learn approaches for pain management and expectations for surgical recovery from partnerships with God. Furthermore, reliance on medications was often minimized due to fear of harmful side effects. In particular, we note that patients had little knowledge of the existence and purpose of opioids. The fear of side effects and limited understanding of opioids may have prevented these patients from pursuing pharmacologic pain management, potentially resulting in under-treatment. We note that these same factors may have enabled patients to develop stronger coping methods and mechanisms of resilience when faced with painful experiences. These findings suggest that individuals’ values, beliefs, and past experiences contribute greatly to their management of pain. During clinical care, health care providers should be encouraged to gain a thorough understanding of a patient’s pain coping behaviors and identify strategies to support those coping mechanisms when appropriate. This approach is especially important for clinicians working with patients in the developing world as well as minority patients in the developed world. Achieving adequate pain relief may be challenging or ineffective, if not introduced in the context of patients’ individual coping mechanisms and resources. While Dominican patients’ experiences may not represent the pain experience of patients of other diverse backgrounds, we emphasize the value of the patient-physician partnership in achieving appropriate pain relief. For certain populations, such a partnership may limit the use of opioids while ensuring that patients bring adequate resources to bear against disease stressors including pain. This strategy may also help minimize the risk of side effects, addiction, and diversion from opioid use [8, 9].

Several limitations of our study should be noted. Our sample size was comprised of OpWalk Boston patients, who represent a small sample of the individuals with arthritis pain in the Dominican Republic. We acknowledge they may not be typical of the larger population of persons with arthritis pain, as they actively sought pain relief through TJR. Furthermore, due to timing constraints, interviews were performed within a span of four days, limiting our capacity to incorporate accumulating knowledge to our interview protocol as data were collected. Finally, our analysis may have misinterpreted subtle aspects of language. To minimize this, we worked verbatim from interview text and involved Dominican clinicians during the analysis process in order to ensure objective and culturally appropriate interpretations of transcripts.

## Conclusions

In conclusion, many patients reported modest use of pharmacologic therapies, and the majority employed non-pharmacologic therapies, including religion, to manage arthritis pain. Patients felt equipped to cope with postsurgical pain and persevere through recovery. Understanding patients’ individual coping approaches and resources in developing countries will enable health care professionals to better determine optimal pain management interventions for patients of diverse backgrounds. In particular, physicians should identify pain management plans that are congruent with patients’ cultural beliefs and coping mechanisms. Consideration of these coping strategies could be employed in reducing the burden of chronic arthritis. The approach could result in less reliance on powerful analgesics including opioids, particularly for patients of diverse backgrounds who employ alternative pain coping mechanisms and do not traditionally use strong medications to manage pain. We also suggest that lessons from Dominican patients’ conceptions of pain could be applied to the development of pain management pathways in developed countries and international mission programs such as OpWalk. Management of chronic arthritis pain could be improved if tailored to a patient’s preferred coping strategies.

## Ethics approval and consent to participate

Consent for participation was obtained from all patients. All study activities were approved by the institutional review board at Brigham and Women’s Hospital.

## Consent for publication

Consent for publication was obtained from all patients.

## Availability of data and materials

The data supporting the conclusions of this article are included within the manuscript and in Table 2. Given the quantity of qualitative text transcribed from the interviews, the original interview transcripts from this study will not be shared on a public database.

## Abbreviations

OpWalk: operation walk boston
TJR: total joint replacement
